# X-rays Stimulate Granular Secretions and Activate Protein Kinase C Signaling in Human Platelets

**DOI:** 10.3390/cimb45070380

**Published:** 2023-07-19

**Authors:** Muhammad Shoaib Khan, Chunliang Liu, Fanbi Meng, Mengnan Yang, Kangxi Zhou, Renping Hu, Xuexiang Wang, Kesheng Dai

**Affiliations:** Jiangsu Institute of Hematology, The First Affiliated Hospital and Collaborative Innovation Center of Hematology, State Key Laboratory of Radiation Medicine and Protection, Medical College, Soochow University, Key Laboratory of Thrombosis and Hemostasis, Ministry of Health, Suzhou 215006, China; khanmuhammadshoaib207@gmail.com (M.S.K.); lcl1986@suda.edu.cn (C.L.); mmengfanbi@163.com (F.M.); ymn9602@163.com (M.Y.); zhoukx2590@163.com (K.Z.); hurenping@suda.edu.cn (R.H.); xxwang62656@suda.edu.cn (X.W.)

**Keywords:** X-rays, platelets, granular secretions, apoptosis, PKC signaling

## Abstract

X-rays can induce morphological as well as functional changes in cells. Platelets are anuclear cellular fragments originating from megakaryocytes and are the major regulators in hemostasis and thrombosis. Platelet products are irradiated to avoid medical complications associated with platelet transfusion. So far, gamma, UV, and laser radiation have been used for this purpose. However, scientists are divided about the effects of radiation on platelet quality. The present study was designed to explore the possible effects of X-rays in washed human platelets and understand the molecular mechanism behind them. In the present study, we exposed washed human platelets to 10 or 30 Gy X-rays at 0.25 Gy/min. Flow cytometry, aggregometry, and western blot were performed to investigate the effect of X-rays on platelet degranulation, integrin activation, platelet aggregation, and apoptosis. It was found that X-rays immediately induced granular secretions with no effect on GP IIb/IIIa activation. Not surprisingly, due to granule secretions in irradiated platelets, platelet aggregation was significantly reduced. In contrast to granular secretions and platelet aggregation, X-rays induced mitochondrial transmembrane potential depolarization in a time-dependent manner to induce apoptosis and activated protein kinase C (PKC) signaling. This study revealed and explained the molecular mechanism activated by X-rays in washed human platelets. Here we also introduced Gö 6983, a PKC inhibitor, as an agent that counteracts X-ray-induced changes and maintains the integrity of platelets.

## 1. Introduction

The discovery of X-rays by Wilhelm Conrad Rontgen gave birth to modern Radiobiology. X-rays are high-energy ionizing radiation that can induce morphological and functional changes in healthy, as well as tumor, cells [1]. It is well documented that ionizing radiation affects cells by damaging their DNA directly or indirectly [2,3]. However, depending on the nature and dose of radiation, cells do respond to ionizing radiation. Platelets are anuclear cellular fragments generated from megakaryocytes and are the primary regulators in the hemostasis and thrombosis [4]. Platelet products are routinely irradiated to avoid medical complications associated with platelet transfusion [5]. Therefore, it is important to understand the impact of radiation on platelets. So far, gamma rays, UV, or laser irradiation have been used in most studies to understand the effects of radiation on platelets. However, the results of these studies are not consistent. Most scientists claim that gamma radiation has no immediate negative effect on platelets’ function, quality, and lifespan [6,7]. However, it is also a fact that gamma rays induce free radicals generation, which can be fatal for platelet quality and function during storage [8]. Similarly, the use of UV radiation has been found to be more destructive to platelet quality and can lead to apoptosis upon storage for a long time [9,10,11,12]. Despite these adverse effects, it is still thought that neither gamma nor UV rays have any immediate effect on platelet quality and are considered safe. Furthermore, a low rate of laser irradiation has been reported to have an anti-aggregative effect on platelets when incubated with agonists [13,14]. Such types of mixed ideas are still circulating about the influence of radiation on platelets. Even a recent study claims that both X-rays and gamma have no harmful effects on platelets quality [15]. Although these results are noteworthy but can still lead one to believe that platelets are highly resistant to ionizing radiation, although platelets are very sensitive anuclear cellular fragments. As we know that ionizing radiation causes cell death in nucleated cells [16]. This indicates the lethal nature of ionizing radiation. Similar to nucleated cells, platelets also undergo apoptosis under different physiological conditions and treatments [17]. Therefore, one can imagine that ionizing radiation can also induce apoptosis in human platelets. However, it is not yet fully understood how radiation induces apoptosis in human platelets. Therefore, the current study was designed to solve this dilemma. We exposed washed human platelets to 10 Gy X-rays for 40 min and 30 Gy for 2 h at a very low rate of 0.25 Gy/min and then examined their effects on platelet degranulation, integrin activation, platelet aggregation, and apoptosis. 

In the present study, we showed that X-rays immediately induced CD62P exposure and ATP release from alpha and dense granules, respectively. Due to granule secretions in irradiated platelets, we found that platelet aggregation was significantly reduced when stimulated with agonists. Furthermore, we reported that X-rays activated mitochondria-mediated platelet apoptosis in a time-dependent manner. To add further, X-rays induced degradation of anti-apoptotic Bcl-xL and upregulation of pro-apoptotic Bak, which resulted in caspase-3 activation. Here we found that X-rays activated Protein Kinase C (PKC) signaling to enhance apoptosis. PKC is a large family of serine–threonine kinase involved in many cellular processes directly or indirectly [18]. There are many isoforms of PKC responsible for diverse functions in the platelets [19]. Here, for the first time, we introduce Gö 6983, a PKC inhibitor, as an agent that can effectively balance radiation-induced changes and maintain the integrity of platelets. We showed that Gö 6983 has the ability to inhibit X-ray-induced CD62P exposure, ΔΨm depolarization, and rescue platelet aggregation. The present study provides a complete picture of platelets’ behavior when exposed to X-rays at 0.25 Gy/min.

## 2. Materials and Methods

### 2.1. Antibodies and Reagents

FITC-conjugated anti-human CD62P antibody (304904) was purchased from Biolegend (San Diego, CA, USA). FITC-conjugated PAC-1 (340507) was purchased from BD Biosciences (San Jose, CA, USA). JC-1 (C2005) and Fluo-4 AM (S1060) were purchased from the Beyotime Institute of Biotechnology (Beyotime, Shanghai, China). FITC-conjugated lactadherin (BLAC-FITC) was purchased from Haematologic Technologies (Essex Junction, VT, USA). ADP (384), Collagen (385), Thrombin (386), ATP Standard (387), and Luciferin/luciferase reagents (395) were purchased from Chrono-log Corporation (Havertown, PA, USA). U46619 (538944) was purchased from Calbiochem (La Jolla, CA, USA). PAGE Gel Fast Preparation Kit (PG 113) was purchased from Yazin Biotechnology (Shanghai, China). Antibodies for western blot against β-Actin (4970), GAPDH (2118), caspase-3 (9662), Bcl-xL (2764), Bak (D2D3) (6947), P53 (7F5) (2527), phospho-P53 (Ser46) (2521), phospho-VASP (Ser157) (3111), PKC α (2056), PKC δ (2058), Akt (9272), and phospho-Akt (Thr308) (13038) were purchased from Cell Signaling Technology (Beverly, MA, USA).

### 2.2. Washed Human Platelets Preparation

Washed human platelets were obtained as described previously [20]. Briefly, whole blood was collected from healthy volunteers and anti-coagulated with 1:7 volume of acid citrate dextrose (ACD: 2.5% trisodium citrate, 2.0% D-glucose, 1.5% citric acid). The whole blood was centrifuged at 1100 rpm for 11 min to collect platelet-rich plasma (PRP). To get washed platelets, PRP was centrifuged at 3500 rpm for 2 min and washed the pellet twice with citrate glucose sodium chloride buffer (CGS: 0.123 M NaCl, 0.033 M D-glucose, 0.013 M trisodium citrate, pH 6.5). Human washed platelets were finally resuspended in modified Tyrode’s buffer (MTB: 2.5 mM Hepes, 150 mM NaCl, 2.5 mM KCl, 12 mM NaHCO_3_, 5.5 mM D-glucose, 1 mM CaCl_2_, 1 mM MgCl_2_, pH 7.4) to a final concentration of 3 × 10^8^/mL. Human washed platelets were allowed to rest for 1–2 h at room temperature, as described previously [21]. Sysmex XP-100 Hematologic Analyzer (Sysmex Corporation, Kobe, Japan) was used to count platelets. 

### 2.3. X-rays Treatment

X-RAD 320ix cabinet X-rays irradiator (PXi, Madison, USA) with an irradiation area of 20 cm × 20 cm at 50 cm SSD was used. Washed human platelets were divided into a control group (non-irradiated) and an irradiated group. In the irradiated group, platelets were exposed to 10 Gy X-rays for 40 min and 30 Gy for 2 h at an irradiation rate of 0.25 Gy/min with a uniform voltage of 320 KVp in a 6-well plate. The radiation exposure was performed at room temperature.

### 2.4. Flow Cytometric Analysis

Washed human platelets were exposed to 10 or 30 Gy X-rays at 0.25 Gy/min. Platelet activation was determined by CD62P exposure and GP IIb/IIIa activation. CD62P expression was detected with FITC-labelled anti-human CD62P (200 μg/mL) antibody (1:5), while GP IIb/IIIa activation was detected by FITC-labelled PAC-1 binding (25 μg/mL). Similarly, for apoptosis detection, mitochondrial inner transmembrane potential (ΔΨm) depolarization and PS exposure were detected by JC-1 (2 μg/mL) and FITC-labelled lactadherin (10 μg/mL), respectively. Gallios™ Flow Cytometer (Beckman Coulter, Brea, USA) was used to measure the effect of X-rays on platelet activation and apoptotic markers at different time points [22]. Similarly, flow cytometric analysis was also performed to detect the elevation in Calcium (Ca^2+^) concentration using Fluo-4 AM (2 mM). Briefly, 5 µL platelets were suspended in 50 µL MTB. Then, 2 µL Fluo-4AM was added and incubated for 20 min at room temperature. After incubation, 300 µL MTB or PBS was added to stop the reaction, and flow cytometry was performed to collect results. 

### 2.5. ATP Release Assay

To examine the effect of X-rays on ATP release from dense granules in washed human platelets, luciferin/luciferase reagents were used in the absence or presence of agonists, as described previously [23]. Firstly, washed human platelets (3 × 10^8^/mL) were exposed to 10 or 30 Gy at 0.25 Gy/min. After X-rays treatments, an ATP release assay was performed at different time points using luciferin/luciferase reagents in the absence of an agonist to determine ATP secretion. These time points were counted from the end of irradiation. For ATP quantification and calibration, an ATP standard was made. Secondly, we also checked ATP secretion in response to agonist (thrombin, collagen, or U46619) stimulation in control or irradiated platelets at 1–2 h after X-ray treatment.

### 2.6. Platelet Aggregation

Washed human platelets (3 × 10^8^/mL) were exposed to 10 or 30 Gy X-rays at 0.25 Gy/min. One hour later, X-ray-irradiated human platelets and non-irradiated platelets were stimulated with thrombin, collagen, U46619, or ADP. A Chrono-Log aggregometer was used to record platelet aggregation at a stirring rate of 1200 rpm at 37 °C. Platelet aggregation was monitored over 8–10 min [24].

### 2.7. Western Blotting

To identify the apoptotic pathway induced by X-rays, cell lysate was prepared, and a western blot was performed. Platelets were lysed with 5× lysis buffer on ice for 15 min. Proteins were separated using 12.5% gel, and ECL Chemiluminescence System was used to visualize on Care stream X-ray Film. Quantification was performed with Image J software (Java 1.8.0.172).

### 2.8. Gö 6983 Incubation

To investigate the effect of Gö 6983 on X-ray-induced CD62P exposure and ΔΨm depolarization, washed human platelets were pretreated with 10 nM Gö 6983 for 2 h before X-ray exposure. Then Gö 6983 human platelets were exposed to 10 or 30 Gy X-rays, using the same rate and time. Flow cytometry or aggregometry were performed to determine the effect of Gö 6983 on X-ray platelet activation, platelet aggregation, or apoptotic markers. 

### 2.9. Statistical Analysis

Data were analyzed using Graph Pad Prism 8 software. All data were presented as mean ± SD. Results were compared with the controls at the corresponding time points. Numeric data were analyzed using One-Way or Two-Way ANOVA. Differences were considered significant at *p* < 0.05.

## 3. Results 

### 3.1. X-rays Immediately Induce CD62P Exposure 

**Figure 1:**
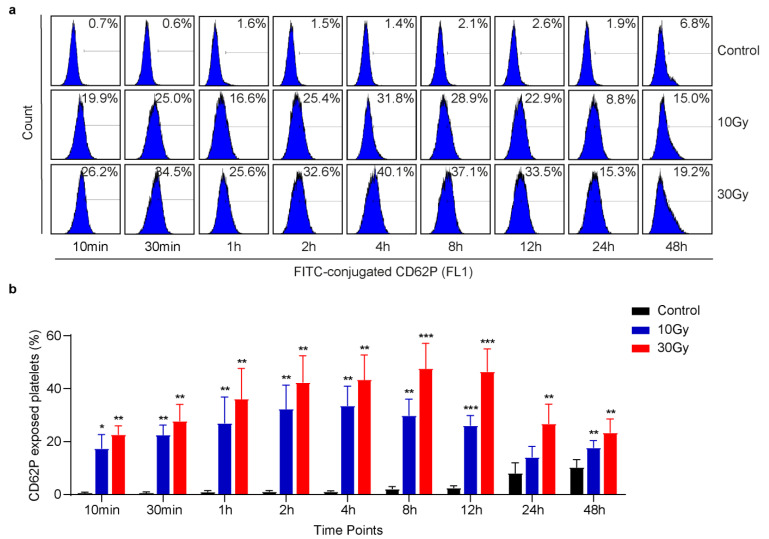
X-rays immediately induce CD62P exposure in washed human platelets. Washed human platelets were exposed to 10 or 30 Gy X-rays at 0.25 Gy/min. (**a**) Representative flow cytometric figures of CD62P exposure. (**b**) Quantification of CD62P exposure induced by X-rays; n = 5. Data are expressed as mean ± SD, * p < 0.05, ** *p* < 0.01, *** *p* < 0.001 compared with control by Two-Way ANOVA followed by Tukey’s multiple comparison tests.

To investigate the effect of X-rays on platelet activation, washed human platelets were exposed to 10 or 30 Gy X-rays at 0.25 Gy/min, and then flow cytometric analysis was performed at different time points [20]. Normally, platelet activation is characterized by CD62P exposure or GP IIb/IIIa activation. It was found that X-rays immediately induced CD62P exposure in human platelets, as shown in Figure 1. The results also showed that X-ray-induced CD62P exposure was dose-dependent. The quantification analysis further revealed a steady increase and then decrease in CD62P exposure in a time-dependent manner (Figure 1b). Similar to CD62P, GP IIb/IIIa is another platelet activation marker. FITC-conjugated PAC-1 was used to detect GP IIb/IIIa activation in irradiated platelets. In contrast to CD62P, we did not observe integrin activation, as shown in Supplementary Figure S1a,b. These data strongly suggest that X-rays induce CD62P release from alpha granules to activate platelets with no effect on GP IIb/IIIa activation.

### 3.2. X-rays Induce ATP Release in Washed Human Platelets

**Figure 2:**
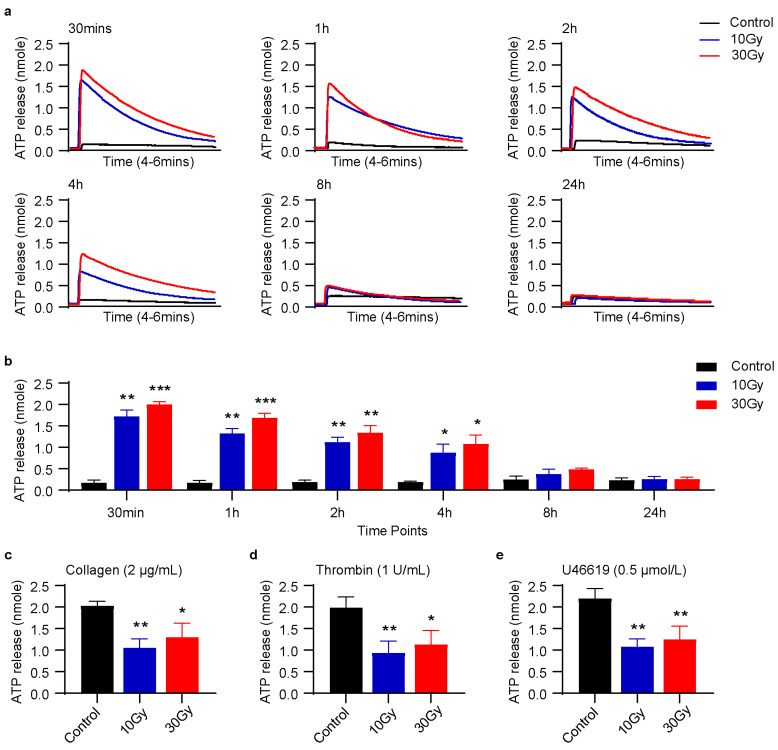
X-rays induce ATP release in washed human platelets. Washed human platelets were exposed to 10 or 30 Gy X-rays at 0.25 Gy/min. ATP release assay was performed at using luciferin/luciferase reagents. (**a**) Original traces of ATP release at different time points without agonist incubation. (**b**) Quantification of ATP release at different time points without agonist incubation. (**c**–**e**) Quantification of ATP release in response to collagen, thrombin, or U46619 incubation after X-rays treatment; n = 4. Data are expressed as mean ± SD, * p < 0.05, ** p < 0.01, *** p < 0.001, compared with control by Two-Way (**a**,**b**) or One-Way ANOVA (**c**–**e**) followed by Tukey’s and Dunnett’s multiple comparison tests, respectively.

A strong agonist is an agent which induces platelet activation, as well as ATP release from dense granules [25]. So far, we found that X-rays immediately induced CD62P release from alpha granules (Figure 1). Therefore, we speculated that X-rays might act as an agonist and might induce ATP release from dense granules as well, although it has been previously reported that even gamma irradiation had no adverse effect on platelet quality and release responses [26]. However, in the case of nucleated cells, it has been found that ionizing radiation can induce ATP release [27]. Our findings showed that X-rays acted as an agonist or activator and immediately induced ATP release from dense granules using luciferin/luciferase reagents without any agonist stimulation in washed platelets (Figure 2a,b). This confirmed ATP secretion in irradiated platelets. It was further noticed in Figure 2a,b; ATP release was higher at the initial time points and then steadily decreased over time. To further verify our results, it was hypothesized that if X-rays immediately induced ATP release, then incubation of agonists may not have any significant effect on ATP release. As expected, when X-ray-irradiated and control platelets were stimulated with agonists, ATP release in irradiated platelets was significantly lower than in control platelets, as shown in Figure 2c–e. The control platelets were at a resting state; the addition of agonists activated them, and ATP was released from dense granules. While irradiated platelets were already activated, therefore, agonist stimulation did not exert an obvious effect on ATP release. The obtained results strongly supported our prediction that X-rays induced ATP release from dense granules in washed human platelets.

### 3.3. X-rays Significantly Reduce Platelet Aggregation

**Figure 3:**
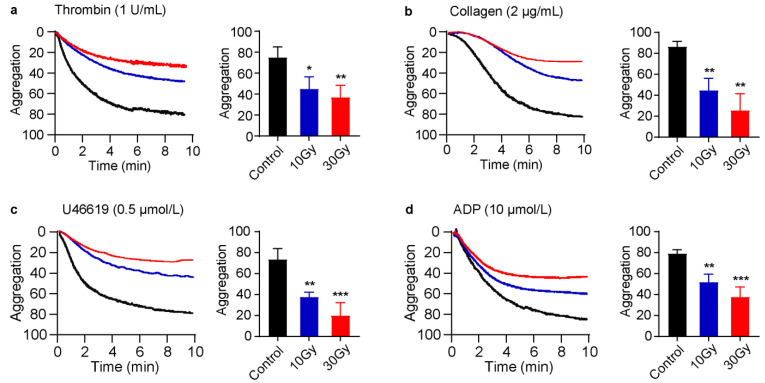
X-rays significantly inhibit platelet aggregation. Washed human platelets were exposed to 10 or 30 Gy X-rays at 0.25 Gy/min. An hour later, irradiated or non-irradiated platelets were incubated with (**a**) thrombin, (**b**) collagen, (**c**) U46619, or (**d**) ADP at constant stirring. Platelet aggregation was monitored over 8–10 min. Data are expressed as mean ± SD obtained from three independent experiments. * p < 0.05, ** p < 0.01, *** p < 0.001, compared with control by One-Way ANOVA followed by Dunnett’s multiple comparison test.

One of the primary functions of platelets is aggregation [28]. Previously, it is reported that ionizing radiation has no adverse effect on platelet aggregation [29]. In Figure 1 and Figure 2, we found that X-rays immediately induced alpha as well as dense granules secretions; therefore, we hypothesized that X-rays might also affect platelet aggregation. An hour after X-ray treatment, both irradiated and non-irradiated platelets were stimulated with thrombin (1 U/mL), collagen (2 µg/mL), U46619 (0.5 µmol/L), or ADP (10 µmol/L). Not surprisingly, platelet aggregation was significantly reduced in 10 and 30-Gy-irradiated platelets, as presented in Figure 3. None of the agonists induced normal aggregation in irradiated platelets. Furthermore, it was observed that the anti-aggregative effect induced by X-rays was also dose-dependent. Based on the obtained results, it was concluded that X-rays significantly suppressed platelet aggregation due to granular secretions.

### 3.4. X-rays Induce Apoptosis in Human Platelets in a Time-Dependent Manner

**Figure 4:**
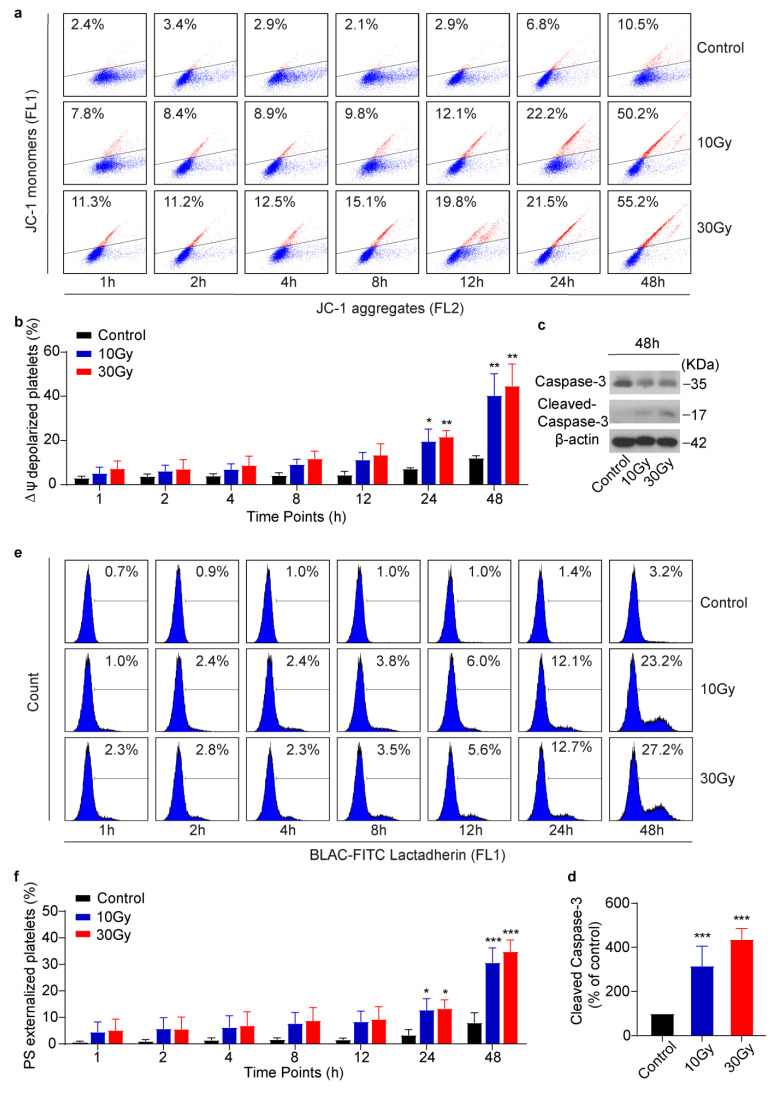
X-rays induce platelet apoptosis in washed human platelets. (**a**) Representative flow cytometric figures of ΔΨm depolarization. (**b**) Quantification of platelets ΔΨm depolarization; n = 4. (**c**) Representative western blot figure of caspase-3. (**d**) Quantification of cleaved caspase-3; n = 3. (**e**) Representative flow cytometric figures of PS externalization. (**f**) Quantification of PS externalization; n = 4. Data are expressed as mean ± SD; * p < 0.05, ** p < 0.01, *** p < 0.001, compared with control by One-Way (**c**,**d**) or Two-way ANOVA (**a**,**b**,**e**,**f**) followed by Bonferroni’s multiple comparison test.

Studies have shown that X-rays can induce apoptosis in cancer cells [30], while, according to the literature, most groups agree that ionizing radiation has no adverse on platelets quality. To investigate the effect of X-rays on platelet apoptosis, flow cytometric analysis and western blot were performed [22]. Flow cytometric analysis revealed that, in contrast to CD62P exposure, X-rays did not have an immediate effect on mitochondrial inner transmembrane potential (ΔΨm) depolarization. Interestingly, the effect became very evident in irradiated platelets at a 48-h time point. The results obtained suggested that X-ray-irradiated platelets entered the apoptotic process 48 h later due to obvious ΔΨm depolarization, as shown in the dot plots in Figure 4a. It has been reported that ΔΨm depolarization is followed by many other apoptotic events, such as caspase-3 activation [31]. Therefore, to confirm whether X-rays activated caspase-3, a western blot was performed. It was found that caspase-3 was activated in irradiated platelets (Figure 4c,d). It is well known that caspase-3 is a primary apoptotic executioner [32]. To investigate further, it has been seen that caspase-3 activation is often followed by PS externalization in platelets [33]. Flow cytometric analysis proved that X-rays induced PS externalization at a 48-h time point as well (Figure 4e,f). It is worth noting that X-rays induced no significant effect on ΔΨm depolarization or PS exposure at the initial time points. These results suggested that X-rays shortened platelets’ lifespan by inducing apoptosis via ΔΨm depolarization, caspase-3 activation, and PS externalization in a time-dependent manner.

### 3.5. X-rays Induce Rearrangement of Bcl-2 Family Proteins and Activate PKC Signaling

**Figure 5:**
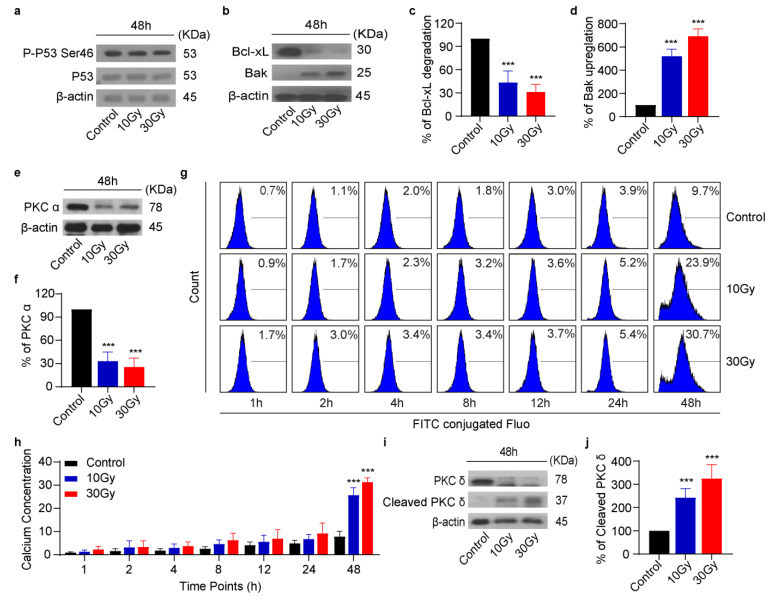
X-rays induce rearrangement of Bcl-2 family proteins and activate PKC signaling. (**a**,**b**) Representative western blot figures of P53, Bcl-xL, or Bak. (**c**) Quantification of Bcl-xL degradation; n = 3. (**d**) Quantification of Bak upregulation; n = 3. (**e**) Representative western blot figure of PKC α. (**f**) Quantification of PKC α; n = 3. (**g**) Representative flow cytometric figures. (**h**) Quantification of rise in Ca^2+^ concentration; n = 3. (**i**) Representative western blot figure of PKC or cleaved PKC δ. (**j**) Quantification of cleaved PKC δ; n = 3. Data are expressed as mean and ± SD, *** p < 0.001, compared with control by One-Way (**a**–**f**,**i**,**j**) or Two-Way ANOVA (**g**,**h**) followed by Dunnett’s or Tukey’s multiple comparison tests, respectively.

From Figure 4, we found that X-rays induced apoptosis in washed human platelets in a time-dependent manner. Next, we aimed to identify the apoptotic mechanism activated by X-rays. From western blot studies, it was found that X-rays induced no effect on P53 expression in human platelets (Figure 5a). Normally, when cells are exposed to ionizing radiation, they undergo apoptosis by the activation of P53 signaling. Further, we attempted to study the effect of X-rays on Bcl-2 family protein expression. Western blot results indicated that X-rays induced degradation of anti-apoptotic Bcl-xL and upregulation of pro-apoptotic Bak in platelets, as given in Figure 5b–d. The degradation of Bcl-xL and upregulation of Bak lead to caspase-3 activation (Figure 4c,d). It has been previously reported that Bcl-xL degradation and Bak upregulation facilitated the release of cytochrome C to activate caspase-3 to induce apoptosis [34]. It is well-known that once caspase-3 is activated, it affects other regulatory proteins [35]. Here we found that PKC signaling was activated in irradiated platelets. X-rays significantly reduced PKC α expression at 48 h (Figure 5e,f). Since PKC α activity is highly sensitive to Ca^2+^ concentration, flow cytometric studies established that X-rays induced a significant elevation in Ca^2+^ concentration in a time-dependent manner (Figure 5g,h). Furthermore, similar to PKC α, PKC δ is another prime isoform of PKC signaling. Western blot analysis proved that X-rays activated PKC δ and generated active cleaved PKC δ fragment (37 KDa), as shown in Figure 5i,j. Reduction in the total content of PKC α and δ clearly suggested that PKC signaling is activated in irradiated platelets to induce apoptosis.

### 3.6. Gö 6983 Inhibits X-rays Induced CD62P Exposure, ΔΨm Depolarization and Rescues Platelet Aggregation

**Figure 6:**
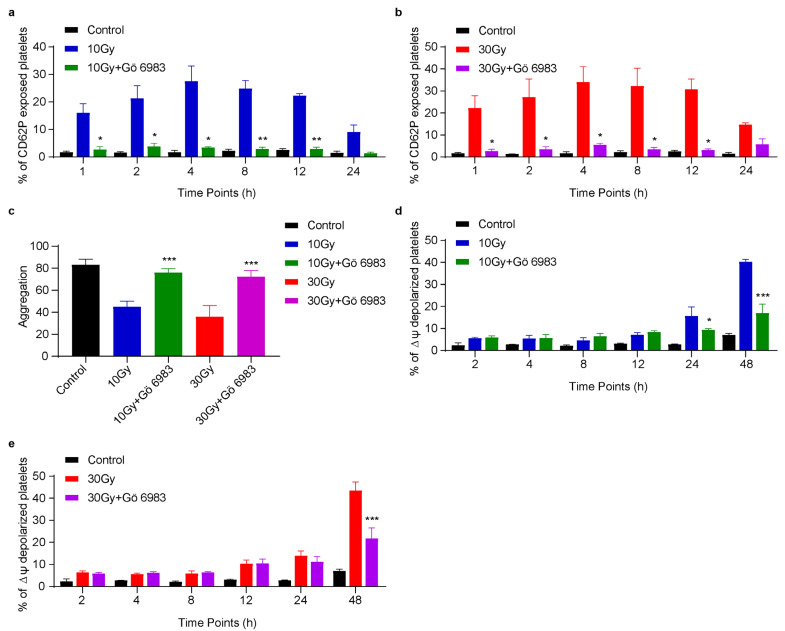
Gö 6983 inhibits X-ray-induced CD62P and ΔΨm depolarization and rescues platelet aggregation. Washed human platelets were pretreated with 10 nM Gö 6983 for 2 h with gentle shake and then exposed to 10 or 30 Gy X-rays at 0.25 Gy/min. (**a**,**b**) Quantification of 10 Gy or 30 Gy induced CD62P exposure without or with Gö 6983 incubation; n = 3. (**c**) Quantification of platelet aggregation without or with Gö 6983 incubated irradiated platelets when stimulated with thrombin; n = 3. (**d**,**e**) Quantification of 10 Gy or 30 Gy induced ΔΨm depolarization without or with Gö 6983 incubation; n = 3. * p < 0.05, ** p < 0.01, *** p < 0.001, compared irradiated platelets to Gö 6983 incubated irradiated platelets by One-Way (**c**) or Two-Way (**a**,**b**,**d**,**e**) ANOVA.

Since X-rays induced granular secretions and apoptosis, we pretreated washed human platelets with 10 nM Gö 6983 for 2 h before X-ray exposure to minimize radiation-induced changes. Gö 6983 is an inhibitor for a wide range of PKC isoforms [36]. We noticed that CD62P exposure was significantly inhibited in Gö 6983 incubated irradiated platelets compared to irradiated platelets without Gö 6983 incubation (Figure 6a,b). While CD62P exposure in non-Gö 6983 incubated irradiated platelets followed the same trend as Figure 1. The results showed that this inhibitor was effective against both doses of X-rays. Additionally, we found that Gö 6983 successfully rescued platelet aggregation and ensured platelets’ functional integrity (Figure 6c). We further postulated that Gö 6983 might also inhibit X-ray-induced platelet apoptosis. Flow cytometric analysis confirmed that ΔΨm depolarization was inhibited in Gö 6983 incubated irradiated platelets compared to irradiated platelets without Gö 6983 incubation, as given in Figure 6d,e. This suggested that Gö 6983 carries the potential to inhibit X-ray-induced platelet apoptosis as well.

## 4. Discussion

In the current study, we discovered the critical impact of X-rays on washed human platelets at a low rate of irradiation. First, from flow cytometric analysis, we found that 10 or 30 Gy X-rays significantly induced CD62P release from alpha granules to the plasma membrane. Likewise, some reports showed that gamma or UV-radiation-induced CD62P exposure in a time-dependent manner [37,38,39]. Their studies did not recommend the immediate effect of radiation on CD62P exposure. However, our results confirmed that X-rays induced an instantaneous effect on CD62P exposure in human platelets. It is important to notice that CD62P exposure in platelets can be fatal and may lead to a number of events [40,41,42]. It was also observed that CD62P exposure first increased and then decreased in X-ray-irradiated platelets. The decrease in CD62P exposure might be due to CD62P shedding over time [43]. It is not surprising to know that X-rays induced CD62P exposure in washed platelets; reports have shown that radiation results in the generation of free radicals, such as reactive oxygen species (ROS), which lead to CD62P exposure [44]. Unlike CD62P, we did not observe integrin activation in irradiated platelets even upon thrombin stimulation as well (Supplementary Figure S1a,b). Inactivation of GP IIb/IIIa by X-rays rules out the involvement of inside-out signal transduction. Studies showed that ionizing radiation is found to have no effect on the integrin activation [15]. Normally, integrin activation in platelets depends on incubation with a strong agonist or activator, such as thrombin. However, it is not necessary that both activation markers should respond the same way to a particular stimulus [45]. 

We further aimed to investigate the effect of X-rays on ATP release from dense granules. It is well known that platelets release ATP from dense granules when incubated with a strong agonist [46]. Similar to CD62P release, our results strongly suggested that X-rays acted as an activator and induced ATP release from dense granules without agonist stimulation using luciferin/luciferase reagents at different time points. Since platelet activation is an energy-driven process, ATP release in irradiated platelets fully supports our results [47]. It was further noticed that ATP release continuously decreased with time (Figure 2a,b). This decrease suggests hydrolysis of ATP to ADP or AMP in platelets [48]. However, ATP release response was significantly low in irradiated platelets than in control when incubated with agonists (Figure 2c–e). These results strongly suggested that X-rays induced granular secretions in human platelets.

Next, to discuss the effect of X-rays on platelet function, it was found that platelet aggregation was significantly reduced when 10 or 30 Gy irradiated platelets were stimulated with thrombin, collagen, U46619, or ADP, as shown in Figure 3. As it is understood, GP IIb/IIIa controls the platelet aggregation [49]. Whenever an agonist is incubated with platelets, it activates GP IIb/IIIa and induces aggregation. The flow cytometry results revealed that X-rays induced no effect on GP IIb/IIIa activation, but still, the incubation of agonists with irradiated platelets was unable to activate GP IIb/IIIa to induce normal aggregation. We suggest that the reduction in platelet aggregation in irradiated platelets in response to agonist stimulation was due to granular secretions. Furthermore, the inactivation of integrin also supports the anergic behavior of irradiated platelets (Supplementary Figure S1a,b). To strengthen our results, it has been found that ATP release inhibits platelet aggregation [48]. Similar observations were made when laser-irradiated platelets were incubated with agonists [13,14,50]. To further support our findings, another study reported that gamma radiation could be lethal to platelet function due to the production of free radicals during long-term platelet storage that causes exposure to CD62P and affects platelet function [51].

The literature study shows that ionizing radiation has no negative effect on the human platelet quality [15]. However, here we found that X-rays induced apoptosis in washed human platelets in a time-dependent manner when irradiated at 0.25 Gy/min (Figure 4). X-rays triggered mitochondria-mediated apoptosis by inducing ΔΨm depolarization and caspase-3 activation, followed by PS externalization. Several studies have demonstrated that platelets typically undergo apoptosis by following mitochondrial or intrinsic pathway [52]. To understand the apoptotic mechanism induced by X-rays, first, it was found that X-rays induced ΔΨm depolarization in a time-dependent manner, thereby inducing degradation of anti-apoptotic Bcl-xL and upregulation of pro-apoptotic Bak (Figure 5b–d). The reorganization of these key proteins resulted in caspase-3 activation, which, in turn, led to PS externalization. Similar observations were made when platelet products were treated with UV irradiation and stored for a longer time [10,53]. It is important to note that, normally, in nucleated cells, ionizing radiation activates P53 to induce apoptosis [54]. To regulate apoptosis, P53 is phosphorylated at the serine 46 residue [55]. However, in contrast, we found that X-rays did not induce P53 activation or phosphorylation in human platelets, as shown in Figure 5a. 

It is known that active caspase-3 carries the ability to activate other signaling pathways to enhance apoptosis [35]. Here, we found that the PKC signaling pathway was activated in irradiated platelets. The total content of PKC α and δ was obviously reduced, and even PKC δ resulted in cleaved PKC δ fragments. Reports suggest that the reduction in PKC α content can enhance apoptosis in bladder cancer [56]. Similarly, caspase-3 activation and PS externalization are responsible for PKC δ activation [57,58,59]. Cleaved PKC δ can further enhance apoptosis by translocating to different cell organelles [60,61]. Likewise, results were obtained when human thyroid cells were exposed to ionizing radiation to induce apoptosis [62]. It is well understood that PKC signaling comprises Ca^2+^-dependent and independent pathways [63]. Based on our results, we found that X-rays activated both Ca^2+^-dependent and independent pathways. Once PKC signaling is activated, it can further affect other regulatory pathways, such as AKT, to promote apoptosis [64]. Surprisingly, we found that X-ray-induced PKC activation exerted no effect on AKT activation or p-VASP expression, as shown in Supplementary Figure S2a,b.

Here, we attempted to overcome X-ray-induced CD62P exposure and ΔΨm depolarization to maintain platelet quality to inhibit platelet activation and apoptosis. It was found that Gö 6983 significantly inhibited X-ray-induced CD62P exposure or ΔΨm depolarization and rescued platelet aggregation (Figure 6). It is known that Gö 6983 effectively inhibits the release of peroxides, as well as intracellular Ca^2+^ accumulation, and allows organs to function normally [65,66]. Previously, our lab has also reported that Gö 6983 can inhibit apoptosis in platelets [67]. We demonstrated that Gö 6983 inhibited the release of CD62P from alpha granules and also preserved functional and mitochondrial integrity in platelets by preventing ΔΨm depolarization after X-ray exposure. Based on our findings, the use of Gö 6983 is recommended to suppress radiation-induced damage and maintain the integrity of platelets during storage. However, further research is needed to explore all possible effects of Gö 6983 on platelet quality and integrity, which will be useful during platelet transfusion and storage.

## 5. Conclusions

The present study concluded that human platelets are highly sensitive to X-rays when irradiated at 0.25 Gy/min. We demonstrated that X-rays immediately induced granular secretions, due to which platelet aggregation was significantly reduced. We also found that X-ray-irradiated platelets undergo apoptosis via activation of PKC signaling. Furthermore, Gö 6983 was found to be very effective in inhibiting X-ray-induced platelet activation and apoptosis.

## Data Availability

The data presented in this study are available upon request from the corresponding author.

## Supplementary Materials

The following supporting information can be downloaded at: https://www.mdpi.com/article/10.3390/cimb45070380/s1, Figure S1: X-rays induce no effect on GP IIb/IIIa activation**.** Washed human platelets were exposed to 10 or 30 Gy X-rays at 0.25 Gy/min. Flow Cytometric analysis was performed at different time points with PAC-1 binding. (a) Quantification of PAC-1 positive platelets to determine GP IIb/IIIa activation. (b) Quantification of PAC-1 binding in irradiated platelets upon 0.01 U/mL thrombin. Data are expressed as mean ± SD from three independent experiments. **** p <* 0.001, compared with controls by Two-Way (a) or One-Way ANOVA (b) followed by Tukey’s and Dunnett’s multiple comparison tests, respectively; Figure S2: X-rays induce no effect on AKT or p-VASP. Washed human platelets were exposed to 10 or 30 Gy X-rays at 0.25 Gy/min and western blot was performed 48 h after X-ray exposure. X-rays induced no effect on the expression of (a) AKT and (b) p-VASP. All the experiments were performed in triplicate.
