# Supplementary material for: X-rays Stimulate Granular Secretions and Activate Protein Kinase C Signaling in Human Platelets
Source: Curr Issues Mol Biol. 2023 Jul 19;45(7):6024–39. doi: 10.3390/cimb45070380 (PMC10378519; doi:10.3390/cimb45070380)
Supplement: Supplementary file 1 [file cimb-45-00380-s001.zip › cimb-2431299-supplementary.pdf]

Supplementary Figure S1

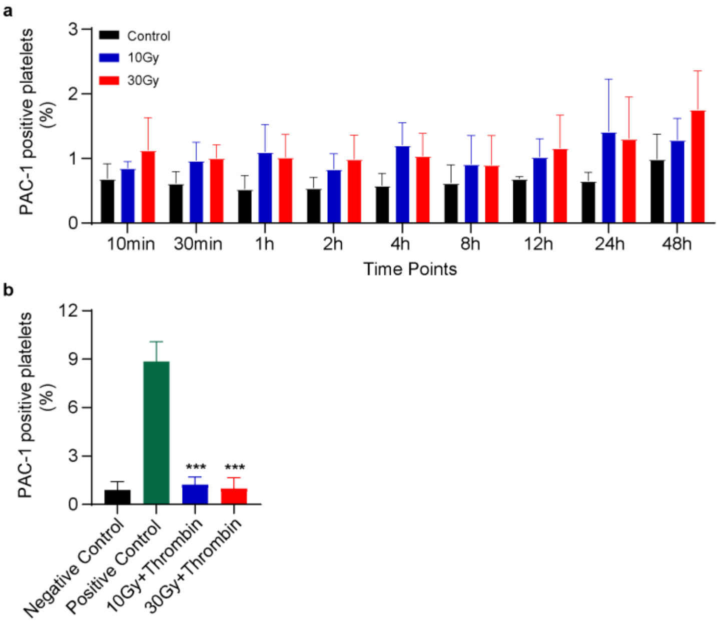

Figure S1. X-rays induce no effect on GP IIb/IIIa activation**.** Washed human platelets were exposed to 10 or 30Gy x-rays at 0.25Gy/min. Flow Cytometric analysis was performed at different time points with PAC-1 binding. (a) Quantification of PAC-1 positive platelets to determine GP IIb/IIIa activation. (b) Quantification of PAC-1 binding in irradiated platelets upon with 0.01U/mL thrombin. Data are expressed as mean ±SD from three independent experiments. *** *P* <*0.001*, compared with controls by Two Way (a) or One Way ANOVA (b) followed by Tukey's and Dunnett's multiple comparisons test respectively.

Supplementary Figure S2

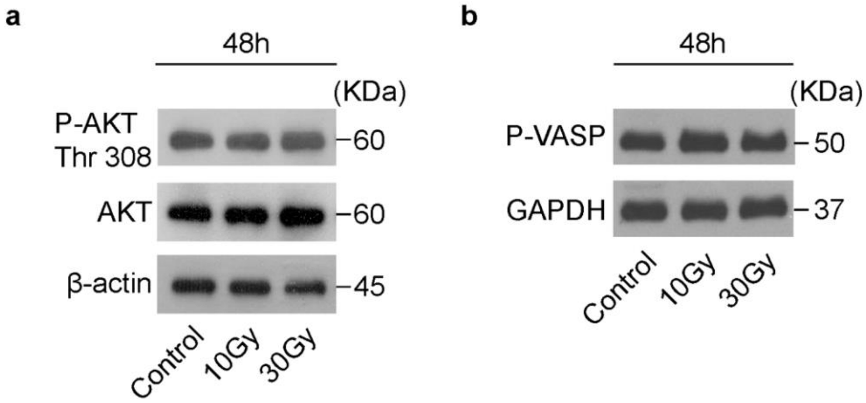

Figure S2. X-rays induce no effect on AKT or p-VASP. Washed human platelets were exposed to 10 or 30Gy x-rays at 0.25Gy/min and western blot was performed 48h later x-rays exposure. X-rays induced no effect on the expression of (a) AKT and (b) p-VASP. All the experiments were performed in triplicate.
